# Seeing Objects as Faces Enhances Object Detection

**DOI:** 10.1177/2041669515606007

**Published:** 2015-09-30

**Authors:** Kohske Takahashi, Katsumi Watanabe

**Affiliations:** Research Center for Advanced Science and Technology, The University of Tokyo, Tokyo, Japan; Department of Intermedia Art and Science, School of Fundamental Science and Engineering, Waseda University, Tokyo, Japan

**Keywords:** Face perception, face detection, face awareness, face inversion effect, signal detection theory

## Abstract

The face is a special visual stimulus. Both bottom-up processes for low-level facial features and top-down modulation by face expectations contribute to the advantages of face perception. However, it is hard to dissociate the top-down factors from the bottom-up processes, since facial stimuli mandatorily lead to face awareness. In the present study, using the face pareidolia phenomenon, we demonstrated that face awareness, namely seeing an object as a face, enhances object detection performance. In face pareidolia, some people see a visual stimulus, for example, three dots arranged in V shape, as a face, while others do not. This phenomenon allows us to investigate the effect of face awareness leaving the stimulus per se unchanged. Participants were asked to detect a face target or a triangle target. While target per se was identical between the two tasks, the detection sensitivity was higher when the participants recognized the target as a face. This was the case irrespective of the stimulus eccentricity or the vertical orientation of the stimulus. These results demonstrate that seeing an object as a face facilitates object detection via top-down modulation. The advantages of face perception are, therefore, at least partly, due to face awareness.

## Introduction

The face is a special visual stimulus for humans; faces are easy to detect, preferentially attended, and hard to ignore (Hershler, Golan, Bentin, & Hochstein, 2010; Hershler & Hochstein, 2005, 2006; Langton, Law, Burton, & Schweinberger, 2008; Purcell & Stewart, 1988; VanRullen, 2006). The brain has a region named the Fusiform Face Area (FFA) that is dedicated to facial processing (Grill-Spector, Knouf, & Kanwisher, 2004; Kanwisher & Yovel, 2006; Kanwisher, McDermott, & Chun, 1997). What makes faces so different from other objects? The bottom-up visual pathway that particularly responds to a facial configuration is critical. Inverted or scrambled faces are less efficiently processed than typical facial configurations (Purcell & Stewart, 1988). The right FFA is preferentially activated by facial patterns irrespective of face awareness (Caldara & Seghier, 2009).

However, there is more to facial perception than bottom-up processes; for example, expectation enhances face and object perception (Esterman & Yantis, 2010; Puri & Wojciulik, 2008), perhaps by constructing appropriate internal templates for the expected inputs (Liu et al., 2014; Smith, Gosselin, & Schyns, 2012) in accordance with the predictive coding theory (Hershler et al., 2010; Hershler & Hochstein, 2005, 2006; Langton et al., 2008; Purcell & Stewart, 1988; Summerfield, Egner, Greene, et al., 2006; Summerfield, Egner, Mangels, & Hirsch, 2006). Imagining a face or predicting the appearance of a face influences the activities of FFA and other relevant regions in both neurotypical individuals (Grill-Spector et al., 2004; Kanwisher & Yovel, 2006; Kanwisher et al., 1997; Mechelli, Price, Friston, & Ishai, 2004; Summerfield, Egner, Greene, et al., 2006) and those with prosopagnosia (Purcell & Stewart, 1988; Righart, Andersson, Schwartz, Mayer, & Vuilleumier, 2010). The FFA also activates even when the presence of a face is implied only contextually (Caldara & Seghier, 2009; Cox, Meyers, & Sinha, 2004). Furthermore, face awareness also matters; while Rubin’s vase gives us the perception of face and the perception of vase stochastically, the neural activities in the face-related regions are modulated depending on whether one is seeing the image as a face or a vase (Andrews, Schluppeck, Homfray, Matthews, & Blakemore, 2002; Esterman & Yantis, 2010; Puri & Wojciulik, 2008; Qiu et al., 2009).

The present study investigated how top-down modulation, in particular face awareness, contributes to the advantages of face perception. Given the preferential responses to typical facial configurations, combined with several forms of top-down modulation on face perception, we can hypothesize two possible accounts. First, facial configuration may be the only factor that determines whether a stimulus is perceived as a face. If FFA serves as a face-pass filter (Caldara & Seghier, 2009; Liu et al., 2014; Smith et al., 2012), the advantages of face perception over the perception of other objects can be explained by the preference for the facial configuration (Purcell & Stewart, 1988). If this were the case, whether a stimulus is seen as a face or not is unimportant. Second, in addition to this purely bottom-up account, we can also hypothesize that face awareness, that is, perceiving that an object is a face, may enhance detection of the object. Practically, this simple question is difficult to address due to the confounding of stimulus configuration and face awareness. Visual inputs of facial patterns mandatorily produce face awareness in observers. In other words, it is very unlikely that observers fail to identify the visual inputs of facial patterns as a face. Furthermore, almost all experimental tasks have explicitly asked participants to search for or detect a face (Hershler & Hochstein, 2005, 2006; Hershler et al., 2010; Langton et al., 2008; Purcell & Stewart, 1988; VanRullen, 2006). Therefore, perceptual performances for face stimuli versus object stimuli involve effects of both the stimulus configuration and awareness of any given configuration as a face. This hinders extracting the specific effect of face awareness on perceptual performance.

To overcome the difficulty of dissociating top-down modulation from bottom-up processes, the present study used the face pareidolia phenomenon. In this phenomenon, objects other than faces are illusorily perceived as a face, for example, a cloud in the sky, the Cydonia region of Mars, or an electrical outlet. Since these pareidolia faces indeed induce face-related neural activities (Bentin & Golland, 2002; Churches, Baron-Cohen, & Ring, 2009; Hadjikhani, Kveraga, Naik, & Ahlfors, 2009), they are essentially processed as a face. Unlike normal faces, however, pareidolia faces do not necessarily lead to face awareness. Individuals sometimes notice the facial configuration of a pareidolia face and see it as a face, yet this is not always the case. Accordingly, contrasts between when a pareidolia face is seen as a face versus when it is not would tell us how face awareness influences face perception, importantly leaving the stimulus per se unchanged. For example, we recently demonstrated that pareidolia faces can produce the gaze cueing effect (Frischen, Bayliss, & Tipper, 2007), but only when observers see the objects as faces (Ristic & Kingstone, 2005; Takahashi & Watanabe, 2013). As such, here we used a pareidolia face as a detection target and tested whether detection performance depends on whether observers saw the target as a face or not.

## Experiment 1

### Methods

#### Participants

Twenty volunteers participated after they gave written informed consent. All of the participants had normal or corrected-to-normal visual acuity. The study was approved by the Ethics Committee of the University of Tokyo and conducted in accordance with the Declaration of Helsinki.

#### Apparatus and stimuli

Participants sat in a dark and quiet room. The visual stimuli were presented on a CRT monitor (the refresh rate was 85 Hz) at a viewing distance of 57 cm. The experiments were presented on an Apple Mac mini with MATLAB and Psychophysics Toolbox extension (Brainard, 1997; Kleiner et al., 2007; Pelli, 1997).

**Figure 1.**
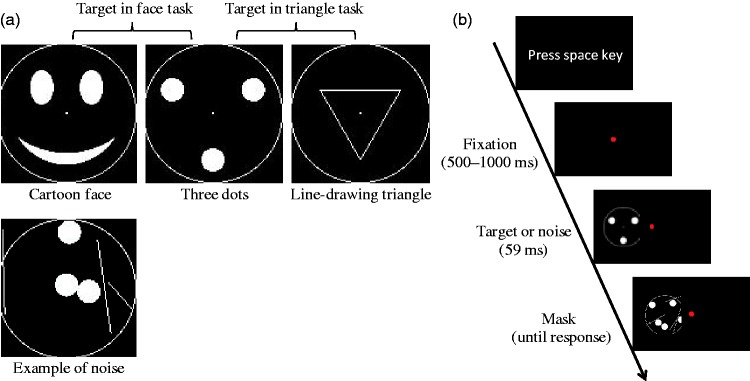
Stimuli and procedure used in Experiment 1. (a) Targets (first row) and an example of noise (second row). The targets on the left and middle were used in the face task, while the targets on the middle and right were used in the triangle task. (b) A trial sequence.

All visual stimuli consisted of a circular frame (radius of 1.55°) with parts inside the circle differing for different stimuli (Figure 1(a)). The cartoon face was composed of a mouth and eyes. The three dots (radius of 0.13°) were arranged in triangle that could be seen as a face or as a triangle. The dots were 1.05° apart from the center of the circle. The vertices of the line-drawing triangle were also 1.05° apart from the center of the circle. A noise stimulus was composed of three dots and three lines. A mask stimulus was composed of five dots (radius of 0.13°) and five lines. The location of dots and lines, as well as the lengths of the lines, were randomly determined for each trial. Stimuli were centered vertically on the screen, while the horizontal position varied from trial to trial; the stimulus appeared on either the left or right side of the screen at one of three eccentricities (2.59°, 5.18°, or 7.76°).

#### Procedure

The participants were randomly assigned to a face task (*N* = 10) or a triangle task (*N* = 10) condition. In the face task, the target stimulus was either the cartoon face or the three dots. Prior to the experiment, the experimenter showed these target stimuli and instructed the participant that the task was to indicate whether the stimulus was a face or a noise. The participants were also told explicitly that both of the target stimuli depicted a face. In the triangle task, the target stimulus was either a line-drawing triangle or the three dots. The participants were told explicitly that both of the target stimuli depicted a triangle. They were also instructed that the task was to indicate whether the stimulus was a triangle or a noise. There was no explicit mention of faces.

Figure 1(b) shows the trial sequence. A trial began by pressing the spacebar. A red fixation dot appeared at the center of screen. Then, after a variable interval (0.5–1 s), a target stimulus or a noise stimulus was presented for 59 ms (five frames), which was followed by the presentation of the mask stimulus until a response was given. The participants were required to press a left-arrow key for face-stimulus or triangle stimulus response and a right-arrow key for noise-stimulus response.

The participants performed 12 familiarization trials with a stimulus duration of 500 ms and then 12 practice trials with a stimulus duration of 59 ms. A main session consisted of 180 noise-stimulus and 180 target-stimulus trials. In the target-stimulus trials, each of six conditions (two target types × three eccentricities) was repeated 30 times. In the noise-stimulus trials, each of three eccentricities was repeated 60 times. The trial sequence was determined in a pseudorandom manner.

#### Data analysis

We calculated d′ as a sensitivity measure and beta as a bias measure based the signal detection theory (Stanislaw & Todorov, 1999; Wright, Horry, & Skagerberg, 2009). Hit rates for the target was estimated independently for the three-dot and the cartoon face or triangle (i.e., nondot) target. It corresponded to the percentage of target responses for each target types. The false alarm rate was common in the calculation of d′ of the three-dot and nondot target and was defined as the percentage of target responses for noise stimulus. This false alarm rate was also used for the calculation of beta. And for beta, hit rate was defined as the percentage of target response for target-stimulus regardless of the target type.

### Results and Discussion

**Figure 2.**
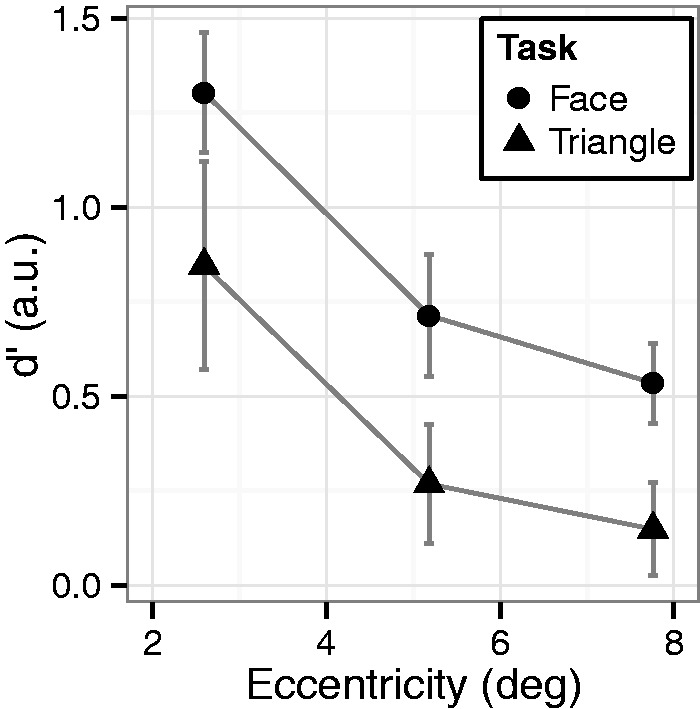
Average d′ in Experiment 1. Error bars indicate SEM.

We calculated the detection sensitivity (d′) for the three-dot target (Figure 2) as well as the criterion or bias (β) based on signal detection theory (Table 1). We performed a two-way mixed ANOVA (task as a between-subject factor and eccentricity as a within-subject factor) on the d′ values for the three-dot target. The d′ in the face task was higher than the d′ in the triangle task (*F*(1, 18) = 5.89, *p* < .05, $\eta_{\text{p}}^{2}$^ ^= 0.25), despite the fact that the target per se was identical between the two tasks. Whereas the peripheral targets were difficult to detect (*F*(2, 36) = 14.3, *p* < .001, η_p_^2 ^= 0.40), the advantage of the face task was observed regardless of the target eccentricity (no interaction of task × eccentricity, *F*(2, 36) = 0.03, *p* = .97, $\eta_{\text{p}}^{2}$^ ^= 0.00). These results clearly demonstrate that seeing objects as faces enhances their detection.

**Table 1.** d′ and β for all Types of Targets in Experiments 1, 2, 3, and 4.

Experiment 1								
Task		Eccentricity	d′ (three-dot)		d′ (non-dot)		β
Face		2.59	1.303	(0.158)	2.612	(0.243)	1.345	(0.387)
		5.18	0.714	(0.161)	2.467	(0.246)	1.406	(0.247)
		7.76	0.534	(0.106)	2.018	(0.230)	1.468	(0.281)
Triangle		2.59	0.846	(0.276)	2.496	(0.351)	1.876	(0.535)
		5.18	0.268	(0.158)	2.005	(0.293)	1.206	(0.139)
		7.76	0.148	(0.123)	1.814	(0.211)	1.165	(0.157)

Experiment 2								
Task	Direction	Eccentricity	d′ (three-dot)		d′ (non-dot)		β
Face	Upright	2.59	1.066	(0.1986)	2.723	(0.3101)	4.181	(1.8762)
		7.76	0.421	(0.1114)	1.758	(0.3201)	2.397	(0.6446)
	Inverted	2.59	0.741	(0.1379)	2.210	(0.2331)	2.452	(1.3550)
		7.76	0.357	(0.1277)	1.339	(0.1892)	1.384	(0.2175)
Triangle	Upright	2.59	0.483	(0.1795)	1.924	(0.2900)	1.009	(0.1120)
		7.76	0.178	(0.1306)	1.397	(0.2554)	1.212	(0.2111)
	Inverted	2.59	−0.015	(0.0995)	2.067	(0.3361)	1.369	(0.1384)
		7.76	−0.144	(0.0958)	1.406	(0.2393)	1.067	(0.1330)

Experiment 3					
Task	Eccentricity	d′ (three-dot)		β	
Face	2.59	0.553	(0.1578)	1.076	(0.0746)
	5.18	0.332	(0.1512)	1.146	(0.0915)
	7.76	0.315	(0.1027)	1.127	(0.0546)
Triangle	2.59	0.565	(0.1706)	1.127	(0.1449)
	5.18	0.404	(0.1552)	1.133	(0.0917)
	7.76	0.125	(0.0745)	0.988	(0.0399)

Experiment 4							
Task	Eccentricity	d′ (diamond)		d′ (non-dot)		β	
Face	2.59	0.5488	(0.1234)	2.8546	(0.2020)	4.0741	(1.2121)
	7.76	0.2441	(0.0933)	1.9600	(0.1899)	1.7184	(0.2653)
Triangle	2.59	0.4753	(0.1230)	2.4001	(0.1802)	1.7174	(0.3659)
	7.76	0.0556	(0.0813)	1.7613	(0.1566)	1.4111	(0.2042)

*Note.* SEM is given in parentheses.

We did not observe any differences in the bias (Table 1; task: *F*(1, 18) = 0.00, *p* = .98, $\eta_{\text{p}}^{2}$^ ^= 0.00; eccentricity: *F*(2, 36) = 1.08, *p* = .35, $\eta_{\text{p}}^{2}$^ ^= 0.06; interaction: *F*(2, 36) = 1.86, *p* = .18, $\eta_{\text{p}}^{2}$^ ^= 0.09). The task type affected only the detection sensitivities and not the false detection of the noise patterns as targets. Furthermore, d′ for the nondot targets were similar across the two tasks (*F*(1, 18) = 0.58, *p* = 0.46, $\eta_{\text{p}}^{2}$^ ^= 0.03), which suggests that the overall task difficulty was comparable.

## Experiment 2

If the facilitation of face detection was based on the successful construction of an internal template of a face (Liu et al., 2014; Smith et al., 2012), the facilitation might be specific to the typical face configuration. In Experiment 2, therefore, we presented upright (V-shaped) and inverted (A-shaped) three-dot stimuli.

### Methods

Twenty-four volunteers were newly recruited. The participants were randomly assigned to the face task (*N* = 12) or the triangle task (*N* = 12). The methods were identical to Experiment 1 except for the following. In Experiment 2, the eccentricity was either 2.59° or 7.76°. The target stimuli in the upright condition were identical to Experiment 1 in the upright condition, that is, the three-dot stimulus and a cartoon face (face task) or a line-drawing triangle (triangle). In the inverted condition, the vertically flipped targets were presented. These two conditions were conducted in separate sessions. The session order was counterbalanced across participants. Participants previewed the target stimuli at the beginning of each session. They then performed 12 familiarization trials, 12 practice trials, and the main session. The main session consisted of 120 noise-stimulus and 120 target-stimulus trials. In the target-stimulus trials, each of four conditions (two target types × two eccentricities) was repeated 30 times. In the noise-stimulus trials, both of two eccentricities were repeated 60 times. The trial sequence was determined in a pseudorandom manner.

### Results and Discussion

**Figure 3.**
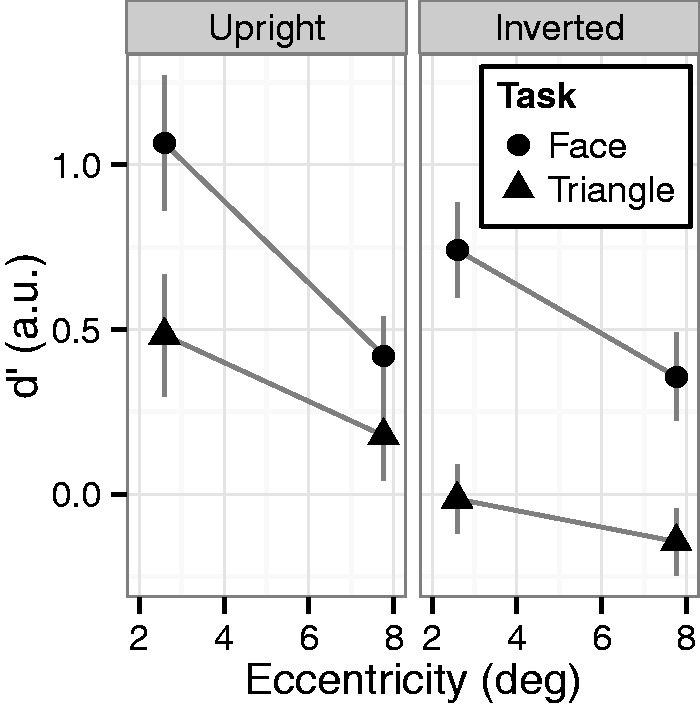
Average d′ in Experiment 2. Error bars indicate SEM.

Figure 3 and Table 1 show the results of Experiment 2. A three-way mixed ANOVA (task as a between factor and eccentricity and vertical orientation as within factors) revealed that, as in Experiment 1, d′ for the three-dot target in the face task was higher than that in the triangle task (*F*(1, 22) = 14.8, *p* < .01, $\eta_{\text{p}}^{2}$^ ^= 0.40). We also observed a face inversion effect; d′ for the upright targets was higher than for the inverted targets (*F*(1, 22) = 11.3, *p* < .01, $\eta_{\text{p}}^{2}$^ ^= 0.34). Importantly, we did not observe a two-way interaction between task and vertical direction (*F*(1, 22) = 1.43, *p* = .22, $\eta_{\text{p}}^{2}$^ ^= 0.06), which suggests that seeing an object as a face enhances object detection even when the object is seen as an inverted face. The absence of this interaction suggests another important implication; in the triangle task, the V-shaped three-dot triangle (upright facial configuration) is easier to detect than the A-shaped three-dot triangle (inverted facial configuration), even if the patterns are not seen as faces (Caldara & Seghier, 2009). The d′ for the nondot targets was comparable between the face and triangle tasks (*F*(1, 22) = 0.82, *p* = .38, $\eta_{\text{p}}^{2}$^ ^= 0.04). Furthermore, we did not find any significant effects regarding bias (task: *F*(1, 22) = 2.63, *p* = .12, $\eta_{\text{p}}^{2}$^ ^= 0.11; eccentricity: *F*(1, 22) = 1.32, *p* = .26, $\eta_{\text{p}}^{2}$^ ^= 0.06; vertical orientation: *F*(1, 22) = 1.91, *p* = .18, $\eta_{\text{p}}^{2}$^ ^= 0.08; task × eccentricity: *F*(1, 22) = 1.15, *p* = .30, $\eta_{\text{p}}^{2}$^ ^= 0.05; task × orientation: *F*(1, 22) = 2.62, *p* = .12, $\eta_{\text{p}}^{2}$^ ^= 0.11; eccentricity × orientation: *F*(1, 22) = 0.04, *p* = .84, $\eta_{\text{p}}^{2}$^ ^= 0.00; three-way interaction: *F*(1, 22) = 1.34, *p* = .26, $\eta_{\text{p}}^{2}$^ ^= 0.06).

## Experiment 3

In the previous experiments, by virtue of face pareidolia, the target stimulus per se was identical between the face task and triangle task. However, we used different nondot targets, namely a cartoon face and a line-drawing triangle, to maintain the observer’s face awareness in the face task. Although we did not find any significant effect regarding bias and sensitivity toward the nondot targets, the presentation of nondot targets might have influenced the detection performance of the three-dot target. For example, previous study showed that nonfacial stimuli induced face-specific brain activity after viewing normally aligned facial stimuli (Bentin & Golland, 2002). Accordingly, instruction to see a stimulus as a face may be insufficient and viewing a face-like stimulus (i.e., the cartoon face) may be necessary to enhance the detection of three-dot stimulus. Furthermore, the results of previous experiment could not rule out the possibility that using the line-drawing triangle as a target may disrupt the detection of three-dot target. Therefore, we decided to conduct control experiments to examine these possibilities. Experiment 3 was a slight modification of Experiment 1; we used only the three-dot target and did not present the cartoon face or the line-drawing triangle. Thus, the purposes and predictions were twofold. First, if viewing a face-like stimulus (i.e., the cartoon face) is necessary to enhance the detection of three-dot stimulus being seen as a face, we should observed the comparable detection performance in the face and triangle task in Experiment 3, which should be lower than that of the face task in Experiment 1. Contrarily, if the instruction to see the three-dot as a face is sufficient to enhance detection, we should observe comparable detection performance between the face tasks in Experiments 1 and 3, which would be higher than the triangle tasks. Second, if the presence of the line-drawing target interfered with the detection of three-dot target in the previous experiments, d′ values for the three-dot target in Experiment 3 would be higher than in Experiment 1.

### Methods

Twenty-two volunteers were newly recruited. The participants were randomly assigned to a face task (*N* = 12) or a triangle task (*N* = 10) condition. The methods were identical to Experiment 1 except for the following. In Experiment 3, only the three-dot stimulus was used as the target. The participants previewed the stimuli and were instructed to detect a face in the face task or a triangle in the triangle task. The main session consisted of 120 noise-stimulus and 120 target-stimulus trials.

### Results and Discussion

**Figure 4.**
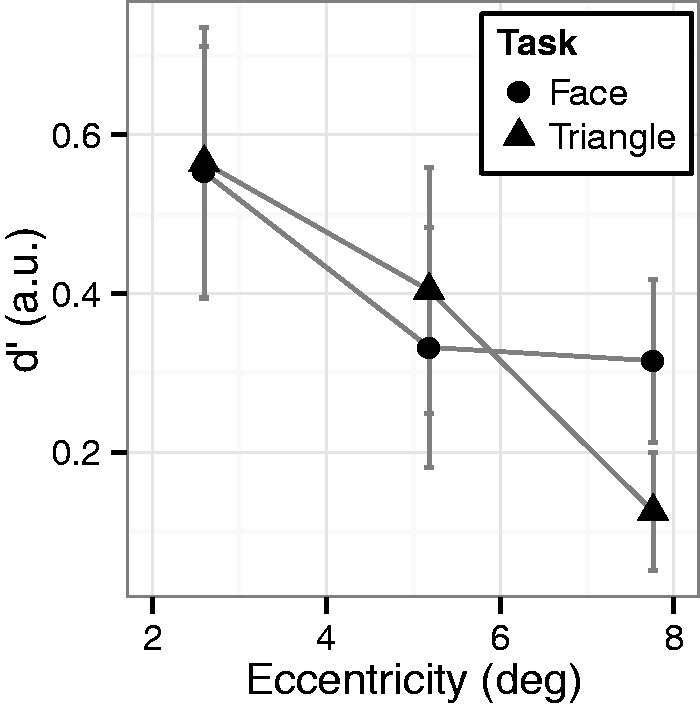
Average d′ in Experiment 3. Error bars indicate SEM.

Figure 4 and Table 1 show the results of Experiment 3. A two-way mixed ANOVA of task and eccentricity revealed the significant main effect of eccentricity (*F*(2, 42) = 5.49, *p* < .01, $\eta_{\text{p}}^{2}$^ ^= 0.21), while the main effects of task (*F*(1, 21) = 0.05, *p* = .83, $\eta_{\text{p}}^{2}$^ ^= 0.00) and interaction (*F*(2, 42) = 0.95, *p* = .40, $\eta_{\text{p}}^{2}$^ ^= 0.04) were not significant. These results suggested that the instruction to see a stimulus, as a face was not sufficient to enhance the stimulus detection. To examine further the effects of the cartoon face, we compared the d′ values of face task in Experiment 3 with those obtained in Experiment 1. A two-way mixed ANOVA of experiment × eccentricity revealed significant main effects of experiment (*F*(1, 21) = 8.08, *p* < .001, $\eta_{\text{p}}^{2}$^ ^= 0.28) and eccentricity (*F*(2, 42) = 10.4, *p* < .001, $\eta_{\text{p}}^{2}$^ ^= 0.33). The interaction almost reached significance (*F*(2, 42) = 3.06, *p* = .057, $\eta_{\text{p}}^{2}$^ ^= 0.13). Thus, the d′ of the face task in Experiment 3 was even lower than that of the face task in Experiment 1 and was comparable with the triangle task in Experiment 3. These results further supported that viewing cartoon face was prerequisite to enhance the detection of three-dot stimulus being seen as a face. We also compared the d′ values of triangle task between Experiments 1 and 3. A two-way mixed ANOVA revealed the significant main effect of eccentricity (*F*(2, 36) = 9.19, *p* < .01, $\eta_{\text{p}}^{2}$^ ^= 0.34), while the main effect of experiment (*F*(1, 18) = 0.09, *p* = .77, $\eta_{\text{p}}^{2}$^ ^= 0.01) and interaction (*F*(2, 36) = 1.22, *p* = .30, $\eta_{\text{p}}^{2}$^ ^= 0.06) were not significant. These results suggested the presentation of line-drawing triangle did not affect the task performances.

The implications of Experiment 3 were twofold. First, the difference between the face task and triangle task in the previous experiments were not due using the line-drawing triangle as a target. Second, the instruction to see the three-dot stimulus as a face was insufficient to enhance the target detection. The enhancement took place only by viewing the normal facial stimulus (i.e., the cartoon face).

## Experiment 4

Experiment 3 highlighted the importance of presentation of a cartoon face. This manipulation might have unexpectedly increased the general vigilance or arousal of participants during the face task. If this was the case, the enhancement of detection would not be specific to face perception, and the detection of any target other than faces should be enhanced. Experiment 4 examined this possibility by replacing the three-dot target by a four-dot diamond target.

### Methods

**Figure 5.**
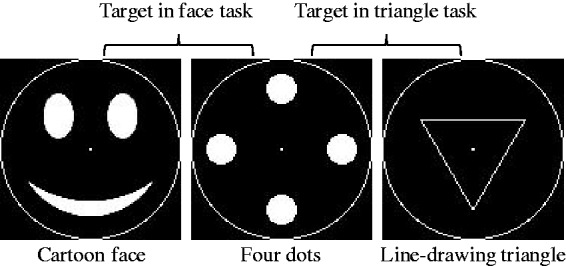
Stimuli used in Experiment 4.

Eighteen volunteers were newly recruited. The methods were identical to those of Experiment 1, except for the following. The participants performed the face task and triangle task in separate sessions. The session order was counterbalanced across participants. In both tasks, the three-dot target was replaced by four dots arranged in a diamond shape (Figure 5). Thus, in the face task, the target was either the cartoon face or the diamond, whereas the line-drawing triangle and the diamond were used as targets in the triangle task. Prior to each session, the participants previewed the target stimuli and were instructed to detect a “face or diamond shape” in the face task and to detect a “triangle or diamond shape” in the triangle task. The stimulus eccentricity was either 2.59° or 7.76°. A main session consisted of 120 noise-stimulus and 120 target-stimulus trials. In the target-stimulus trials, each of four conditions (two target types × two eccentricities) was repeated 30 times. In the noise-stimulus trials, each of two eccentricities was repeated 60 times. The trial sequence was determined in a pseudorandom manner.

### Results and Discussion

**Figure 6.**
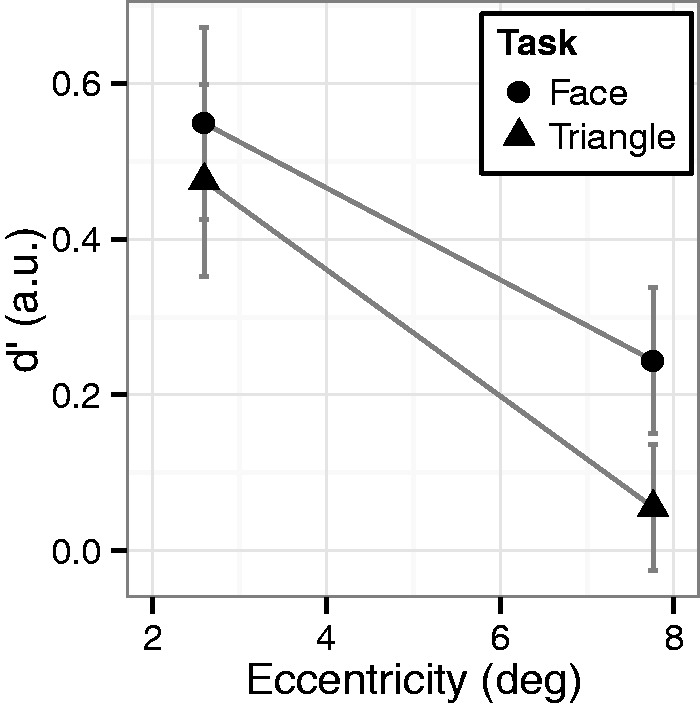
Average d′ for the diamond target in Experiment 4. Error bars indicate SEM.

Figure 6 and Table 1 show the results of Experiment 4. A two-way repeated measures ANOVA on d′ revealed the significant main effect of eccentricity (*F*(1, 17) = 8.76, *p* < .01, $\eta_{\text{p}}^{2}$^ ^= 0.14), while the main effect of task (*F*(1, 17) = 2.00, *p* = .17, $\eta_{\text{p}}^{2}$^ ^= 0.02) and interaction (*F*(1, 17) = 0.49, *p* = .49, $\eta_{\text{p}}^{2}$^ ^= 0.00) was not significant. Although it might be difficult to compare directly between the diamond stimulus in Experiment 4 and three-dot stimulus in Experiment 1, since the stimuli were physically different, the d′ for the diamond stimulus was even lower than that for the three-dot stimulus. Thus, we did not observe any sign that the presentation of the cartoon face enhanced object detection in general via increase of vigilance or arousal.

## General Discussion

The present study investigated whether seeing objects as faces influences visual detection performance. For this, we used face pareidolia as a probe technique and presented novel stimuli that could be perceived as either a face or a triangle. The results showed that detection performance was higher when the target was seen as a face than as a triangle, despite the fact that the target stimulus per se was identical. More specifically, we found that (a) face awareness could enhance detection of stimuli that have a configuration that can be interpreted as a face (i.e., three-dot triangle), and (b) this face awareness is induced and strengthened by the presentation of a cartoon face.

The instruction to see a three-dot target as a face was insufficient to enhance the detection performance (face task in Experiment 3). The enhancement took place only when the participants previewed the cartoon face. Neuroimaging studies have demonstrated that atypical facial stimuli induced the face-specific neural response (N170) only after viewing normally aligned facial stimuli (Bentin & Golland, 2002). Thus, viewing the normally aligned face (the cartoon face in the present study) would strengthen the face awareness for the ambiguous patterns (the three-dot stimulus) when instructed to see them as a face. Then, detection for them would be enhanced.

Careful inspection of the β values, d′ values for the non-dot targets, as well as the results of control experiments, confirmed that the higher d′ for the three-dot target in the face task was not a side effect of the presentation of nondot targets. For example, presentation of line-drawing triangle did not impair the detection (triangle task in Experiment 3). Increased vigilance or arousal by the presentation of cartoon face could not account for the higher d′ (Experiment 4). The enhancement of detection, therefore, truly reflected face awareness induced by viewing a face-like stimulus.

In sum, our findings clearly demonstrated that objects are easier to detect when they are seen as a face than when they are not. While previous studies have repeatedly shown the advantages of face processing versus processing other objects (Hershler et al., 2010; Hershler & Hochstein, 2005, 2006; Langton et al., 2008; Purcell & Stewart, 1988; VanRullen, 2006), none could determine whether bottom-up processes or top-down modulations were primary drivers of the face processing advantage. In contrast, our experimental paradigm allowed us to completely exclude the confounding of low-level feature differences between faces and other objects, simply because the targets were identical. As a result, we obtained unequivocal evidence that face awareness helps object detection.

These results would be consistent with the previous EEG studies showing the top-down modulation on N170 (and some other components, Jemel, Pisani, Rousselle, Crommelinck, & Bruyer, 2005) and correlation with face awareness (Bentin & Golland, 2002; George, Jemel, Fiori, Chaby, & Renault, 2005; Latinus & Taylor, 2005). For example, ambiguous pattern (Mooney face) induced the larger N170 activity after learning to see them as a face (Latinus & Taylor, 2005) or when the pattern was reported as a face (George et al., 2005). Taken together with our findings, seeing something as a face elicits face-specific process and consequently this top-down modulation could enhance detection of face-like patterns.

Expectation enhances perception (Summerfield & Egner, 2009). Expecting faces, houses, or letters facilitates the detection of stimuli from the expected category while hindering the detection of stimuli from unexpected categories (Esterman & Yantis, 2010; Puri & Wojciulik, 2008) by constructing the corresponding internal templates (Liu et al., 2014; Smith et al., 2012). It is certain that our participants in the face task expected that they would see faces. However, expectation alone cannot account for our results, since the participants in the triangle task also expected the triangle. Hence, what we compared was the detection performance between expected faces and expected triangles. In other words, expecting a face has an advantage over expecting a triangle. Perhaps, expecting a face would activate additional face-related process that otherwise remains inactive. This would explain why faces have their privileged perceptual status.

The advantage of seeing objects as faces was observed regardless of the target eccentricity as well as the vertical orientation of the facial target. Regarding eccentricity, the advantages of face detection over detection of other objects have been observed in both foveal and peripheral visual fields (Hershler et al., 2010). While the study of Hershler and colleagues associated the advantages of face detection with the low-level visual features such as spatial frequencies (Halit, de Haan, Schyns, & Johnson, 2006), the present study demonstrated that even if the stimulus per se was identical, top-down modulation—seeing the stimulus as a face—makes detection easier in both foveal and peripheral vision. Thus, the bottom-up perceptual processes cannot fully explain the advantages of face detection in peripheral vision. The inverted facial patterns were also easier to detect when they were seen as a face, which implies that facilitation of detection by top-down modulation is not specific to the typical face configuration. Taken together, the effects of face awareness are quite general; top-down modulation might be independent from where and how the facial patterns appear to us.

However, top-down processes do not fully explain the facial processing advantage; surprisingly, the upright facial configuration was easier to detect than the inverted facial configuration, even when the stimuli were not seen as a face (Experiment 2, triangle task). With careful interpretation, these results are consistent with previous neuroimaging studies. Activation in the right FFA has been shown to be larger when the number of elements in the upper half of the stimulus is greater than the lower half (i.e., in a V-shape pattern), and critically even when these patterns were not seen as a face (Caldara & Seghier, 2009). Perhaps, the FFA can serve as the bottom-up face-pass filter regardless of face awareness. The advantage of face detection would, therefore, arise from both the top-down modulation related to the face awareness or face expectation and the bottom-up characteristics of the facial pattern detector in FFA.

There is insufficient evidence to fully understand the underlying neural mechanisms of bottom-up and top-down face processing—interactions of face configuration and face awareness—nevertheless we will provide some closing speculations here. FFA—especially the right FFA—might serve as a bottom-up facial pattern filter, regardless of an observer’s intentions, expectations, and face awareness (Caldara & Seghier, 2009). Activity in the right FFA to a pure noise signal was greater when observers saw a pareidolia face in the noise (Liu et al., 2014; Zhang et al., 2008). This would be simply because the noise occasionally formed a facial configuration. However, the activation of FFA would be also susceptible to top-down modulations. The face detection task led to greater activity in FFA and other relevant cortical regions than a letter detection task (Liu et al., 2014), which then modulates the response characteristics of the filter, perhaps increasing the signal-to-noise ratio of FFA activities to facial configurations. Further neuroimaging and psychological studies are warranted to reveal how top-down modulation and bottom-up processes interact in face perception; as shown here, face pareidolia is a powerful tool to investigate how face awareness plays a role in face perception.
